# Personality and cardiovascular mortality risk: a multi-cohort analysis in individuals with and without pre-existing cardiovascular disease

**DOI:** 10.1007/s10865-024-00528-9

**Published:** 2024-10-28

**Authors:** Markus Jokela, Laura Pulkki-Råback, Marko Elovainio, G. David Batty, Mika Kivimäki

**Affiliations:** 1 Department of Psychology, University of Helsinki, Helsinki, Finland; 2 Finnish Institute of Health and Welfare, Helsinki, Finland; 3 Department of Epidemiology and Public Health, University College London, London, UK; 4 Brain Sciences, University College London, London, UK; 5 Department of Public Health, University of Helsinki, Helsinki, Finland

**Keywords:** Personality, Health, Cardiovascular, Stroke, Big five personality

## Abstract

This study investigated the associations between personality traits of the Five Factor Model and cardiovascular mortality, with a specific focus on whether pre-existing cardiovascular conditions modified these associations. We used data from 43,027 participants across five cohort studies: Health and Retirement Study (HRS); Wisconsin Longitudinal Study (WLS); National Social Life, Health, and Aging Project (NSHAP); Midlife in the United States (MIDUS); Household, Income, and Labour Dynamics in Australia (HILDA) with a mean age 55.9 years and 6493 individuals with pre-existing cardiovascular disease. We conducted meta-analyses examining conscientiousness, emotional stability, agreeableness, openness to experience, and extraversion in relation to mortality due to coronary heart disease and stroke. During a mean follow-up of 12.1 years, 1620 participants died from coronary heart disease and 454 from stroke. Lower conscientiousness was associated with higher mortality risk from both coronary heart disease (hazard ratio per 1SD = 0.82, 95%CI = 0.75–0.90) and stroke (HR = 0.84, CI = 0.72–0.99). Lower emotional stability predicted increased coronary heart disease mortality (HR = 0.91, CI = 0.85–0.97). The association between conscientiousness and cardiovascular mortality did not differ between individuals with or without baseline cardiovascular conditions. In addition, adjustments for health behaviors and other covariates only slightly attenuated this association. Other personality traits were not associated with cardiovascular disease mortality. Our findings highlight the role of low conscientiousness, and to a lesser extent low emotional stability, in the development and progression of fatal cardiovascular disease through pathways that may extend beyond established health behaviors.

## Introduction

In our paper published in this journal 10 years ago, we used data from two cohort studies—the Wisconsin Longitudinal Study and the Health and Retirement Study—to examine whether personality traits of the Five Factor Model were associated with cardiovascular disease mortality (Jokela et al., 2014c). We separately examined associations with coronary heart disease and stroke mortality, finding that higher conscientiousness was associated with lower mortality rate due to coronary heart disease (HR = 0.74 per 1 standard deviation, CI = 0.67–0.81) and stroke (HR = 0.78, CI = 0.63–0.97). In addition, higher neuroticism was related to higher risk of coronary heart disease (HR = 1.16, CI = 1.04–1.29) but not stroke, while higher extraversion was related to higher risk of stroke (HR = 1.41, CI = 1.10–1.80) but not coronary heart disease. The current study is an update and an extension of our original study.

A recent study with data from six cohort studies examined how personality was associated with incidence of non-fatal stroke (Stephan et al., 2023). Higher conscientiousness was associated with lower stroke incidence (HR = 0.89, CI = 0.85–0.93). There was no association with extraversion, which our study found to be associated with stroke mortality. Higher neuroticism was associated with elevated stroke incidence (HR = 1.15, CI = 1.10–1.20), while our study found higher neuroticism to be associated only with coronary heart disease mortality. There do not appear to be any pooled-cohort studies since our 2014 that would have examined the risk of coronary heart disease incidence or mortality.

One of the unanswered questions arising from these findings on fatal and non-fatal cardiovascular outcomes is whether personality traits are differently related to cardiovascular mortality among individuals with and without pre-existing cardiovascular disease. Psychosocial risk factors may influence disease progression and therefore be particularly important among those with pre-existing health problems (Kivimäki & Steptoe, 2018). For example, in a pooled analysis of 12 cohort studies (Kivimäki et al., 2018), higher job strain was associated with cardiometabolic mortality only among those with pre-existing cardiometabolic illness, but not among those without cardiometabolic illness; this was observed particularly for men. Job strain may act as a trigger for acute cardiovascular events (Kivimäki & Steptoe, 2018).

We examined whether a similar pattern held for personality traits, that is, whether the associations between personality and cardiovascular mortality were stronger among those with baseline cardiovascular illness compared to those without. This would suggest that personality traits have a greater impact on the progression of pre-existing disease than on the initial development of the disease.

## Methods and materials

Participants were from the Health and Retirement Study (HRS; https://hrs.isr.umich.edu); the Wisconsin Longitudinal Study (WLS; https://wls.wisc.edu); the National Social Life, Health, and Aging Project (NSHAP; https://www.norc.org/research/projects/); Midlife in the United States (MIDUS; http://midus.wisc.edu/); and the Household, Income, and Labour Dynamics in Australia (HILDA; https://melbourneinstitute.unimelb.edu.au/hilda) cohort studies. Table 1 shows the descriptive statistics.

**Table 1:** Descriptive statistics

	HILDA	HRS	MIDUS	NSHAP	WLS
Age, mean (SD)	44.0 (18.0)	68.7 (10.5)	46.8 (12.9)	72.5 (7.1)	53.3 (4.3)
Women, % (n)	53.3 (5778)	59.2 (8038)	52.5 (3288)	53.0 (1166)	53.6 (5410)
Follow-up years, mean (SD)	7.6 (2.7)	9.8 (4.1)	23.4 (6.2)	6.0 (1.3)	14.4 (2.6)
CHD mortality rate per 10,000 (n of deaths)	6.6 (57)	82.0 (1108)	18.7 (273)	49.0 (65)	8.0 (117)
Stroke mortality rate per 10,000 (n of deaths)	2.9 (25)	21.2 (286)	6.7 (98)	18.1 (24)	1.4 (21)
n(participants)	10,853	13,616	6259	2200	10,099

Personality traits of extraversion, emotional stability (i.e., reversed neuroticism), agreeableness, conscientiousness, and openness to experience were assessed with the 36-item Five Factor adjective inventory in HILDA (Saucier, 1994); Midlife Development Inventory (Lachman & Weaver, 1998) in MIDUS (25 items), HRS (26 items), and NSHAP (21 items); and a 29-item version of the Big Five Inventory (BFI; John et al., 1991) in WLS.

Mortality data were derived from death registers and/or information gathered in interview fieldwork. Cause of death was determined with ICD codes (I20-I25 and I60-I69) in HILDA, MIDUS, and WLS. In HRS, the information on causes of death were clustered into broader categories for the public data release, so that the category of “heart problems” included coronary heart disease and myocardial infarction, but also other heart conditions such as heart valve problems, congestive heart failure, and rheumatic heart disease. Stroke was included in the same category as cerebral hemorrhage or accident, and hematoma. In NSHAP, the cardiovascular causes of death were categorized as “heart attack/heart condition” and “stroke/cerebrovascular condition”.

Baseline smoking (yes vs. no), physical inactivity (no weekly moderate/vigorous physical activities vs. some weekly moderate/vigorous physical activities), body mass index (BMI; kg/m^2^), heavy alcohol consumption (> 7 weekly units for women, > 14 weekly units for men), and education (primary, secondary, tertiary) were assessed based on self-reported data. Information on baseline coronary heart disease and stroke were based on self-reported diagnosis status. In HILDA, information on stroke was not available, and baseline data for BMI were not from the same study wave as personality data but the study wave following it after 1 year.

### Statistical analysis

Associations between personality and mortality were estimated using Cox’s proportional hazard models. We first fitted the models in each cohort study separately, and then pooled the estimates with random-effect meta-analysis. Participants were censored at the last available interview date or at the end date of the registry-based mortality data. Separate models were fitted for coronary heart disease and stroke mortality. In each study, all participants with data on personality traits, sex, age, and mortality status were included in the analysis. We imputed missing data on baseline covariates (but not personality traits, age, sex, or mortality status) using stochastic regression imputation, that is, multiple imputation with a single imputation.

## Results

**Fig. 1:**
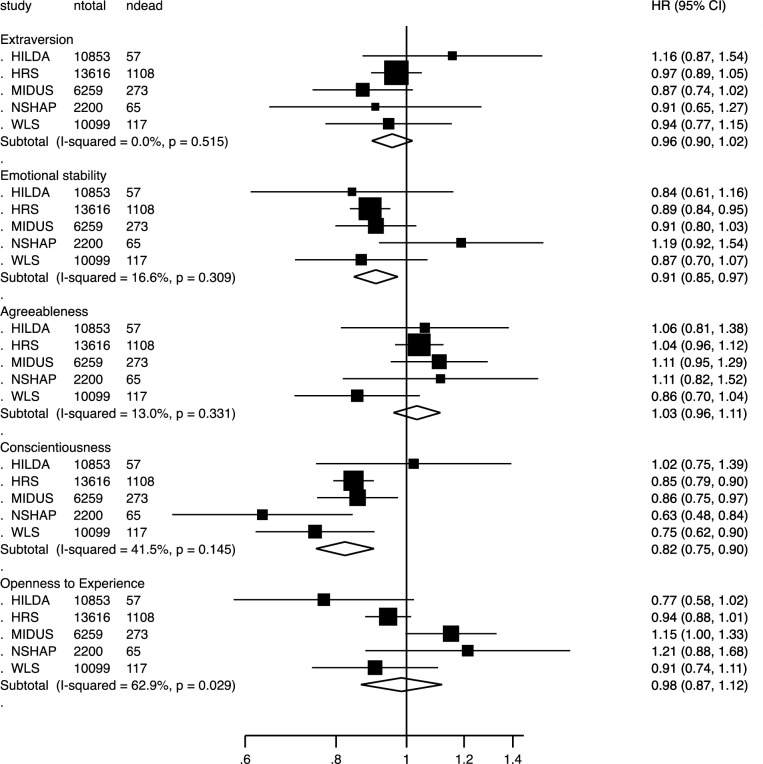
Associations between personality traits and risk of coronary heart disease mortality in a Cox’s proportional hazard model including the five personality traits, age, and sex. Random-effect meta-analysis of 43,027 participants and 1620 deaths. *HRS* Health and Retirement Study; *WLS* Wisconsin Longitudinal Study; *NSHAP* National Social Life, Health, and Aging Project; *MIDUS* Midlife in the United States; *HILDA* Household, Income, and Labour Dynamics in Australia

**Fig. 2:**
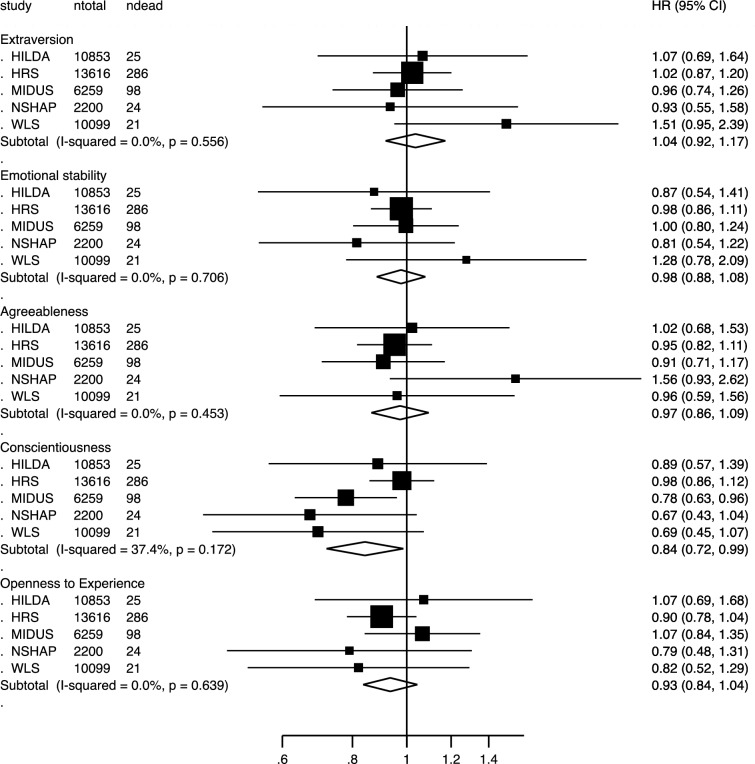
Associations between personality traits and risk of stroke mortality in a Cox’s proportional hazard model including the five personality traits, age, and sex. Random-effect meta-analysis of 43,027 participants and 454 deaths. *HRS* Health and Retirement Study; *WLS* Wisconsin Longitudinal Study; *NSHAP* National Social Life, Health, and Aging Project; *MIDUS* Midlife in the United States; *HILDA* Household, Income, and Labour Dynamics in Australia

There were 1,620 deaths from coronary heart disease and 454 deaths from stroke in the total sample of 43,027 participants (521,363 person-years). Figure 1 shows the personality associations for coronary heart disease mortality, and Fig. 2 for stroke mortality. Coronary heart disease mortality was predicted by lower conscientiousness (HR = 0.82, CI = 0.75–0.90) and lower emotional stability (HR = 0.91, CI = 0.85–0.97), whereas stroke mortality was predicted only by lower conscientiousness (HR = 0.84, CI = 0.72–0.99). When examining mortality due to either coronary heart disease or stroke as a combined outcome, both lower conscientiousness (HR = 0.82, CI = 0.74–0.90) and lower emotional stability (HR = 0.92, CI = 0.88–0.96) were associated with higher cardiovascular mortality risk.

We also calculated the differences in absolute mortality differences. Across the five cohorts, the average rate of coronary heart disease mortality was 30.8 deaths per 10,000 person-years and stroke mortality 8.6 deaths per 10,000 person-years. If we take these rates to describe people with a mean level of conscientiousness, the absolute difference in coronary heart disease death rate for individuals with high conscientiousness (1 standard deviation above the mean) would be 25.3 deaths (= 0.82*30.8) per 10,000 person-years, and the rate for individuals with low conscientiousness (1 standard deviation below the mean) would be 37.6 (= 30.8*(1/0.82) deaths per 10,000 person-years, that is, a difference of 12.4 deaths. The absolute rates for high and low conscientiousness were 7.1 and 10.5 for stroke mortality—a difference of 3.4 deaths per 10,000 person-years. For coronary heart disease mortality of high versus low emotional stability, the differences were 28.0 versus 33.8 deaths per 10,000 person-years.

**Fig. 3:**
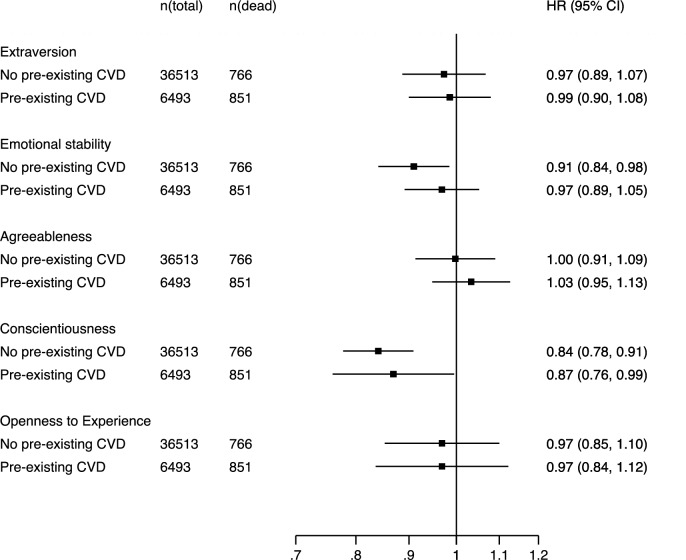
Associations between personality traits and coronary heart disease mortality separately by pre-existing cardiovascular disease (CVD) status at baseline, adjusted for sex and age. Pooled estimates from random-effect meta-analyses of 43,027 participants and 1620 deaths

**Fig. 4:**
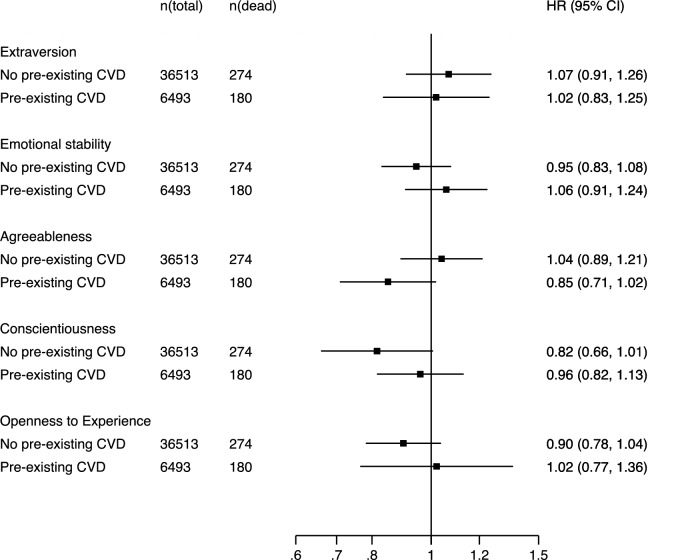
Associations between personality traits and stroke mortality separately by pre-existing cardiovascular disease (CVD) status at baseline, adjusted for sex and age. Pooled estimates from random-effect meta-analyses of 43,027 participants and 454 deaths

Figures 3 and 4 show the pooled associations between personality and cardiovascular mortality separately for participants with and without diagnosed coronary heart disease or stroke at baseline. For coronary heart disease mortality, lower emotional stability was associated with mortality risk only among those without baseline risk (HR = 0.91, CI = 0.84–0.98) but not among those with baseline risk (HR = 0.97, CI = 0.89–1.05), but this difference was not statistically significant (*p* = 0.28 for heterogeneity). The hazard ratios for conscientiousness were very similar for the two groups (0.84 and 0.87; *p* = 0.71 for heterogeneity). For stroke mortality, the association with conscientiousness was stronger among those without baseline risk (HR = 0.82, CI = 0.66–1.02) that those with baseline risk (HR = 0.96, CI = 0.82–1.13), but this difference was not statistically significant (*p* = 0.24 for heterogeneity).

When adjusted for baseline coronary heart disease, stroke, and education, and for smoking, heavy alcohol consumption, physical inactivity, and BMI, the association of conscientiousness with coronary heart disease mortality was attenuated from HR = 0.82 (CI = 0.75–0.90) to HR = 0.91 (CI = 0.85–0.96), and the association with stroke attenuated from HR = 0.84 (CI = 0.72–0.99) to HR = 0.87 (CI = 0.71–1.06). The association of lower emotional stability with coronary heart disease mortality was attenuated completely from HR = 0.91 (CI = 0.85–0.97) to HR = 1.01 (CI = 0.90–1.14) (Table 2).

**Table 2:** Adjusted associations between personality traits and mortality outcomes

	Step 1	Step 2	Step 3
*Coronary heart disease*			
Conscientiousness	0.82 (0.75, 0.90)	0.87 (0.81, 0.94)	0.91 (0.85, 0.96)
Emotional stability	0.91 (0.85, 0.97)	0.96 (0.89, 1.05)	1.01 (0.90, 1.14)
*Stroke*			
Conscientiousness	0.84 (0.72, 0.99)	0.86 (0.71, 1.04)	0.87 (0.71, 1.06)

Values are hazard ratios from random-effect meta-analysis of the five cohort studies. Separate models were fitted for coronary heart disease and stroke. Step 1 inculdes age, sex, and the five personality traits. Step 2 additionally includes baseline coronary heart disease and stroke diagnosis, and education. Step 3 additionally includes physical inactivity, heavy alcohol use, smoking, and body mass index. n = 43,027 participants (1620 coronary heart disease deaths and 454 stroke deaths)

As a sensitivity analysis, we excluded participants who died, or dropped out of follow-up, within 2 years of baseline, as data from these participants might have biased the associations by reverse causation, that is, poor health preceding the impending death influencing personality traits. Conscientiousness was still associated with lower mortality from coronary heart disease (HR = 0.83, CI = 0.77–0.90) and stroke (HR = 0.83, CI = 0.69–1.00), and emotional stability from coronary heart disease (HR = 0.92, CI = 0.87– 0.97), suggesting that reverse causation was unlikely to bias the results considerably.

## Discussion

Our findings consolidate the role of low conscientiousness in predicting elevated risk of cardiovascular mortality, that is, deaths from coronary heart disease and stroke. These associations did not differ by pre-existing cardiovascular disease at baseline, suggesting that low conscientiousness may have a role in both the development and progression of cardiovascular disease. Low emotional stability was associated with coronary heart mortality but not stroke deaths. The updated data analysis indicates that the previously reported association between extraversion and stroke was not robust (Jokela et al., 2014c).

We hypothesized that the association of personality with cardiovascular mortality would be amplified among those with cardiovascular disease at baseline. This was not the case. The vulnerability hypothesis is largely based on the idea that some psychosocial factors, such as stress, can act as triggers for cardiac events rather than a cause of atherosclerosis (Kivimäki & Steptoe, 2018; Kivimäki et al., 2018). Our current findings suggest that the five personality traits examined here do not act as important triggers of cardiovascular events in the same way that psychosocial stress does. Instead, low conscientiousness and low emotional stability are more likely involved in cardiovascular health via other pathways of disease related to initial disease development. Such pathways are likely to result from cumulative factors and experiences over longer time periods (Wagner et al., 2024).

Low conscientiousness has been associated with almost all the poor health outcomes and health behaviors that have been studied, including type-2 diabetes (Jokela et al., 2014b), dementia (Beck et al., 2024), obesity (Jokela et al., 2013), and arthritis (Stephan et al., 2024), among other health outcomes. Low conscientiousness also predicts higher all-cause mortality rate and fewer disability-free life years (Jokela et al., 2020). Cancer risk seems to be the only exception, as cancer risk is not associated with any of the personality traits (Jokela et al., 2014a).

The broad range of associated health outcomes suggests that low conscientiousness is a general upstream risk factor for overall poor health. Intervention and prevention programs that aim to improve people’s health and health behaviors could be more effective if they focused on increasing people’s conscientiousness, behaviors, habits, and cognitive strategies associated with it. This may be more feasible than people often assume. Instead of being stable and unchanging, recent evidence from studies of volitional personality change (Hudson et al., 2020; Rufino et al., 2024) and clinical interventions (Roberts et al., 2017a, 2017b) suggest that personality dispositions can be changed with interventions.

Personality traits have been associated with many of the risky health behaviors that are detrimental to cardiovascular health, including physical inactivity (Sutin et al., 2016), smoking (Hakulinen et al., 2015b), and heavy alcohol use (Hakulinen et al., 2015a). In our original study of cardiovascular mortality (Jokela et al., 2014c), adjustments for health behaviors had almost no effect on the hazard ratios of personality traits. In the study of incident stroke (Stephan et al., 2023), adjusting for baseline health behaviors did not substantially change the associations between personality traits and incident stroke. A similar result was observed in our current study; the adjustments had some effect on the hazard ratios of conscientiousness but did not explain the associations completely. These observations seem to imply that conscientiousness is associated with cardiovascular health via other pathways besides the well-established health behaviors. This would support the idea that the behaviors and cognitions associated with high conscientiousness might be valuable additional components for interventions aimed at improving cardiovascular health. It is, of course, possible that more accurate measurements of health behaviors (e.g., repeated measurements over time) would have explained more of the association between lower conscientiousness and elevated mortality.

In conclusion, the current findings provide further evidence for conscientiousness being the most important personality trait of the Five Factor Model of personality in the development and progression of fatal cardiovascular disease. Pre-existing cardiovascular disease at baseline did not amplify the mortality associations of any personality traits, suggesting that personality may be associated with the long-term development of cardiovascular health, rather than being a trigger of acute cardiovascular events among those with pre-existing cardiovascular condition.
